# Clinical characteristics and IVIG non-responsiveness in Kawasaki disease in infants aged less than 3 months: a retrospective study

**DOI:** 10.3389/fped.2025.1672255

**Published:** 2025-09-26

**Authors:** Cancan Li, Wenqiang Sun, Huawei Wang, Han Zhang, Zhixin Wu, Wenmei Li, Xueping Zhu, Haifeng Geng

**Affiliations:** Department of Neonatology, Children’s Hospital of Soochow University, Suzhou, China

**Keywords:** Kawasaki disease, infant, intravenous immunoglobulin resistance, coronary artery lesions, clinical characteristics

## Abstract

**Objectives:**

To investigate the clinical characteristics of Kawasaki disease (KD) in infants under 3 months of age and to identify risk factors for intravenous immunoglobulin (IVIG) resistance.

**Methods:**

A retrospective analysis was conducted on infants under 3 months with KD hospitalized at the Children's Hospital of Soochow University from January 2020 to December 2024. Patients were divided into complete KD (cKD) and incomplete KD (iKD) groups based on KD diagnostic criteria. Clinical manifestations, CAL incidence, and IVIG responsiveness were compared. Univariate and multivariate logistic regression identified independent risk factors for IVIG resistance.

**Results:**

A total of 47 infants, mean age 70.78 ± 13.55 days, male-to-female ratio 1.47:1, were included. The CAL incidence was 46.81%, with KD onset showing seasonality, peaking in spring and autumn (61.7%). Of the 47 cases, 24 were cKD and 23 iKD. The iKD group had fewer typical symptoms, lower hemoglobin and hematocrit (*P* < 0.05), and higher cardiac troponin T (*P* < 0.05). CAL incidence was higher in iKD (65.2%) compared to cKD (29.2%) (*P* = 0.013), and IVIG resistance was more frequent in iKD group (26.1% vs. 4.2%, *P* = 0.048). The IVIG-resistant group had lower rates of Bacille Calmette-Guérin (BCG) scar reactivation, conjunctival hyperemia, and cough, but exhibited longer fever duration, higher CAL and coronary artery aneurysms (CAA) rates, higher iKD proportion, and elevated PLR and systemic immune-inflammation index (SII) (*P* < 0.05). Multivariate analysis identified PLR ≥ 124.05 as an independent risk factor for IVIG resistance.

**Conclusion:**

Clinical manifestations of KD in infants under 3 months of age are often atypical. Compared with cKD, iKD is associated with a higher risk of coronary artery involvement and IVIG resistance. Elevated PLR may serve as an independent predictor of IVIG resistance in this population.

## Introduction

Kawasaki disease (KD), also known as mucocutaneous lymph node syndrome, is an acute, self-limiting febrile vasculitis of medium- and small-sized vessels with an unknown etiology. It was first described by Tomisaku Kawasaki in the 1960s. KD primarily affects children under 5 years of age and has become the leading cause of acquired heart disease in children in developed countries, with its incidence also increasing annually in developing nations (1). Epidemiological studies have shown that approximately 76%–81% of KD cases occur in children under 5 years old (2, 3). Without timely treatment, around 25% of affected children may develop coronary artery dilation or coronary artery aneurysms, which can lead to severe complications such as myocardial infarction or sudden death (1, 4). Currently, the standard treatment for KD consists of intravenous immunoglobulin (IVIG) combined with aspirin, which can reduce the incidence of coronary artery lesions (CAL) from approximately 25% to about 5%, thereby significantly improving patient outcomes. Nevertheless, 4%–20% of patients may still develop CAL within 6 weeks of disease onset despite receiving IVIG treatment, indicating heterogeneity in the disease's pathogenesis and treatment response (5).

In infants younger than 3 months, the immaturity of the immune system leads to distinct clinical manifestations and immune responses of KD compared to older children. In this age group, classic clinical features such as prolonged fever, conjunctival injection, rash, and lymphadenopathy are often absent or less pronounced, making misdiagnosis as common infections or viral febrile illnesses more likely, which consequently delays diagnosis and treatment. Previous studies have demonstrated that this subgroup is at an increased risk of developing CAL, particularly giant coronary artery aneurysms (1). Mechanistically, the limited humoral immune function and reduced pro-inflammatory cytokine production in young infants may result in a relatively mild systemic inflammatory response, while persistent localized vascular inflammation contributes to endothelial injury and vascular remodeling, ultimately leading to coronary artery damage (6).

It is noteworthy that intravenous immunoglobulin (IVIG) non-responsiveness has been recognized as an independent risk factor for the development of CAL in KD. Approximately 10%–20% of KD patients exhibit poor response to initial IVIG therapy, significantly elevating the risk of adverse cardiovascular outcomes (1). However, in infants younger than 3 months, the mechanisms underlying IVIG resistance remain poorly understood, and reliable clinical predictors have yet to be systematically identified. To address this gap, we conducted a retrospective analysis of KD patients under 3 months of age who were hospitalized at the Children's Hospital of Soochow University over the past five years. This study aims to comprehensively characterize their clinical features, coronary artery involvement, and independent risk factors for IVIG non-responsiveness, thereby providing valuable insights for early identification of high-risk patients and optimization of therapeutic strategies.

## Materials and methods

### Study subjects and methods

This was a retrospective study. The study population comprised infants younger than 3 months who were hospitalized and diagnosed with KD at the Children's Hospital of Soochow University between January 2020 and December 2024. The study was approved by the Ethics Committee of the Children's Hospital of Soochow University. The guardians of all the patients also gave informed consent.

Clinical data of all eligible patients were retrospectively reviewed. According to the diagnostic criteria for KD, patients were categorized into complete KD (cKD) and incomplete KD (iKD) groups. The clinical characteristics, incidence of CAL, and IVIG responsiveness were compared between the groups. Based on the response to IVIG treatment, patients were further divided into IVIG-responsive and IVIG-resistant groups. Clinical characteristics were compared between these two groups, and univariate and multivariate analyses were conducted to identify independent risk factors for IVIG resistance.

### Diagnostic, inclusion, and exclusion criteria

The diagnostic criteria for cKD, iKD, IVIG resistance, and CAL were based on the 2024 guidelines of the American Heart Association (AHA) (1). Inclusion criterias: (1) infants younger than 3 months diagnosed with KD and hospitalized at the Children's Hospital of Soochow University between January 2020 and December 2024; (2) first diagnosis of KD; (3) complete clinical data available. Exclusion criterias: (1) patients who had received IVIG and/or corticosteroids prior to admission; (2) initial IVIG dosage of 1 g/kg or irregular dosing regimens; (3) history of congenital heart surgery or rheumatic diseases; (4) confirmed presence of malignancies, hematologic disorders, genetic metabolic diseases, or other immune-mediated inflammatory diseases.

### Data collection and follow-up

Clinical data were collected, including age at onset, sex, season of onset, clinical features (such as fever, extremity changes, BCG site reactivation, rash, conjunctival hyperemia, oropharyngeal changes, cervical lymphadenopathy, cough, vomiting, diarrhea, nasal congestion and rhinorrhea). Laboratory tests obtained at admission prior to IVIG administration were recorded, including white blood cell count (WBC), absolute neutrophil count (ANC), neutrophil percentage (NEU%), mean platelet volume, platelet count (PLT), hemoglobin (Hb), alanine aminotransferase (ALT), albumin (ALB), cardiac troponin T (cTn-T), creatine kinase-MB (CK-MB), and C-reactive protein (CRP). Inflammatory indices were calculated based on these parameters, including neutrophil-to-lymphocyte ratio (NLR), platelet-to-lymphocyte ratio (PLR), lymphocyte count multiplied by 10,000 divided by CRP (LCR), systemic immune-inflammation index (SII = platelet count × neutrophil count/lymphocyte count), and CRP-to-albumin ratio (CAR). These parameters were analyzed accordingly.

All patients were followed up through a combination of online consultations and outpatient clinic visits to monitor coronary artery lesions dynamically.

### Statistical analysis

Statistical analysis was performed using SPSS version 26.0. Categorical variables were expressed as frequency (percentage) [*n* (%)], and comparisons between groups were performed using the *χ*² test or Fisher's exact test. For continuous variables, non-normally distributed data were analyzed using the Mann–Whitney *U*-test, with results presented as median (interquartile range) [M (P25, P75)], and the test statistic denoted by Z. Normally distributed data were compared using Student's *t*-test, with results expressed as mean ± standard deviation (x¯ ± s). Univariate analyses were conducted to identify significant intergroup differences, and multivariate logistic regression analysis was used to determine independent risk factors. Receiver operating characteristic (ROC) curves were constructed to evaluate the predictive performance of NLR, PLR, and SII for IVIG resistance in infants with KD. A *p*-value < 0.05 was considered statistically significant.

## Results

### General characteristics of infants with KD

Between January 2020 and December 2024, a total of 2,462 children with KD were hospitalized in our center, among whom 48 were younger than 3 months, accounting for 1.95% of all KD cases. After excluding one patient with primary immunodeficiency combined with congenital hypothyroidism, 47 infants were finally enrolled, representing 1.91% (47/2,462) of all KD cases. Among them, 24 cases were classified as cKD and 23 cases as iKD. There were 28 males (59.6%) and 19 females (40.4%), with a male-to-female ratio of 1.47:1. The median length of hospital stay was 11 days (interquartile range: 9–16 days), and the median duration of fever was 6 days (5–7 days), with a median duration of fever at diagnosis of 5 days (4–6 days). The median time to initial IVIG administration was 5 days (4–6 days). Of the 47 patients in this study, all the children received IVIG treatment. Among them, 7 (14.9%) were IVIG-resistant and required IVIG retreatment, along with steroids, which were typically administered after IVIG. The remaining patients did not receive steroids or a second dose of IVIG. All patients with IVIG-resistant were treated with aspirin and either dipyridamole or clopidogrel.

KD cases were observed throughout all seasons, with the highest incidence in spring (March to May) accounting for 34.0% (16/47), followed by autumn (September to November) with 27.6% (13/47), and winter (December to February) and summer (June to August), each accounting for 19.1% (9/47). Regarding clinical manifestations, excluding fever, oropharyngeal changes were the most common symptom (78.7%), followed by rash in 35 cases (74.4%), conjunctival hyperemia in 30 cases (63.8%), cervical lymphadenopathy in 25 cases (53.1%), peripheral limb alterations in 22 cases (46.8%), and BCG site reactivation in 21 cases (44.7%). Perianal erythema or desquamation was noted in 5 cases (10.6%). Respiratory and gastrointestinal symptoms were also common: cough in 41 cases (87.2%), diarrhea in 23 cases (48.9%), nasal congestion and rhinorrhea in 22 cases (46.8%), and vomiting in 7 cases (14.8%). Hepatic dysfunction was present in 13 cases (27.6%). Coronary arteries were normal in 25 patients (53.2%), while CAL was identified in 22 cases (46.8%), and mild pericardial effusion was observed in 2 cases. In addition, cerebrospinal fluid examinations were performed in 8 patients, among whom one was diagnosed with aseptic meningitis and one with facial nerve palsy.

### Comparison of clinical characteristics between cKD and iKD groups

The clinical characteristics of the cKD and iKD groups are summarized in Table 1. Of the 47 patients, 24 (51.0%) were classified as cKD and 23 (48.9%) as iKD. No significant differences were found between the two groups regarding sex, season of onset, hepatic dysfunction, rash, perianal erythema/desquamation, cough, nasal congestion and rhinorrhea, vomiting, diarrhea, or duration of fever at diagnosis (*P* > 0.05). The incidences of BCG site reactivation, conjunctival hyperemia, peripheral limb alterations, cervical lymphadenopathy, and oropharyngeal changes were significantly lower in the iKD group compared to the cKD group (*P* < 0.05). However, the incidences of CAL, steroid usage rate, and coronary artery aneurysm (CAA) were significantly higher in the iKD group than in the cKD group (*P* < 0.05). Of the 47 patients, the incidence rate of CAA is 31.9% (15/47), 13 cases occurred within the first 10 days, and 2 cases occurred after 10 days. Among the 15 cases, there were 11 cases of small aneurysms (Z-score ≥2.5–<5), 2 cases of medium aneurysms (Z-score ≥5 to <10, and absolute dimension <8 mm), and 2 cases of large or giant aneurysms（Z-score ≥10 or absolute dimension ≥8 mm）. In the cKD group, 3 cases developed CAA, while in the iKD group, there were 12 cases. Ten patients (21.3%) developed CAA in the bilateral coronary arteries, 3 (6.4%) developed CAA in the left coronary artery, and 2 (4.3%) developed CAA in the right coronary artery (RCA). The locations of CAA included the left anterior descending artery (LAD) in 11 (23.4%), the left circumflex in 5 (10.6%), the proximal RCA in 12 (25.5%), the middle RCA in 6 (12.8%), and no distal RCA. Patients in the iKD group were younger and had lower Hb and HCT levels but exhibited a longer time to initial IVIG administration, longer fever duration, and higher cTn-T levels, all with statistically significant differences (*P* < 0.05). The incidence of IVIG resistance was also significantly higher in the iKD group compared to the cKD group (*P* < 0.05).

**Table 1:** Comparison of clinical characteristics and laboratory indicators between the cKD group and iKD group [*n* (%)].

Characteristics	cKD group (*n*=24)	iKD group (*n*=23)	*P-value*
Age (days)	79 (69.3, 83.8)	67 (60, 74)	0.016
Sex			
Male	15	13	0.676
Female	9	10	
Season of onset	A	B	0.404
Spring	7	9	
Summer	6	3	
Autumn	5	8	
Winter	6	3	
Hepatic dysfunction	6 (25%)	7 (30.4%)	0.677
Rash	20 (83.3%)	15 (65.2%)	0.154
BCG scar reactivation	16 (66.7%)	5 (21.7%)	0.002
Oropharyngeal changes	23 (95.8%)	14 (60.9%)	0.003
Conjunctival hyperemia	21 (87.5%)	9 (39.1%)	0.001
Peripheral limb alterations	17 (70.8%)	5 (21.7%)	0.001
Cervical lymphadenopathy	17 (70.8%)	8 (34.8%)	0.013
Perianal erythema/desquamation	2 (8.3%)	3 (13%)	0.666
Cough	21 (87.5%)	20 (87%)	1.000
Nasal congestion and rhinorrhea	13 (54.2%)	9 (39.1%)	0.302
Vomiting	3 (12.5%)	4 (17.4%)	0.701
Diarrhea	10 (41.7%)	13 (56.5%)	0.308
Coronary artery lesion	7 (29.2%)	15 (65.2%)	0.013
Coronary artery aneurysm	3 (12.5%)	12 (52.2%)	0.004
IVIG resistance	1 (4.2%)	6 (26.1%)	0.048
Timing of initial IVIG administration (days)	5 (4, 6)	6 (5, 7)	0.018
Steroids	1 (4.2%)	6 (26.1%)	0.048
Duration of fever (days)	5 (4, 6)	7 (6, 8)	0.001
Days of fever at diagnosis	5 (4, 6)	5 (4, 6)	0.299
White blood cell count (×10^9^/L)	13.67 ± 5.27	14.64 ± 4.39	0.497
Hemoglobin (g/L)	99 (93.75, 107.50)	92 (84, 103)	0.020
Mean platelet volume (fl)	9.50 ± 1.19	9.86 ± 1.20	0.245
Absolute neutrophil count (×10^9^/L)	7.75 (5.51, 9.78)	7.82 (4.78, 9.52)	1.000
Neutrophil percentage (%)	55.95 ± 12.02	54.60 ± 13.26	0.716
Platelet count (×10^9^/L)	394.04 ± 142.69	447.22 ± 185.24	0.275
NLR	1.81 (1.15, 2.33)	1.67 (1.00, 2.37)	0.537
PLR	105.08 (65.60, 131.65)	107.41 (58.61, 130.11)	0.782
SII (×10^9^)	658.71 (491.55,1,007.42)	610.49 (39,610, 176.8)	0.966
Hematocrit (L/L)	0.303 ± 0.030	0.286 ± 0.027	0.040
C-reactive protein (mg/L)	76.58 ± 43.11	82.38 ± 55.17	0.686
Alanine aminotransferase (U/L)	23 (17.65, 30.53)	20.8 (16.5, 44.2)	0.725
Albumin (g/L)	39.04 ± 2.71	38.38 ± 2.75	0.416
Prealbumin (mg/L)	79.5 (64.25, 89.25)	82.0 (55.0, 92.0)	0.741
CAR	1.95 ± 1.09	2.20 ± 1.54	0.717
Cardiac troponin T (pg/ml)	13.77 (9.88, 22.79)	21.45 (16.89, 27.43)	0.010
Creatine kinase-MB (U/L)	1.87 (1.37, 2.67)	2.40 (1.62, 4.00)	0.118
LCR	638.33 (321.30, 1,052.27)	557.42 (310.26, 1,799.47)	0.789

cKD, complete Kawasaki disease; iKD, incomplete Kawasaki disease; BCG, Bacille Calmette-Guérin; IVIG, intravenous immunoglobulin; NLR, neutrophil-to-lymphocyte ratio; PLR, platelet-to-lymphocyte ratio; SII, systemic immune-inflammation index; LCR, lymphocyte count multiplied by 10,000 divided by CRP; CAR, C-reactive protein-to-albumin ratio.

### Risk factors for IVIG resistance

The comparison of clinical characteristics between the IVIG-responsive and IVIG-resistant groups is shown in Table 2. Among the 47 patients, 40 (85.1%) were IVIG responders, and 7 (14.9%) were non-responders. No significant differences were observed between the two groups regarding age and sex (*P* > 0.05). Similarly, no significant differences were found in the incidence of hepatic dysfunction, rash, oropharyngeal changes, peripheral limb alterations, cervical lymphadenopathy, perianal erythema/desquamation, nasal congestion and rhinorrhea, vomiting, or diarrhea (*P* > 0.05).

**Table 2:** Comparison of clinical characteristics and laboratory parameters between IVIG-responders and IVIG non-responders [n (%)].

Characteristics	IVIG resistance group (*n*=7)	IVIG-response group (*n*=40)	*P-value*
Age (days)	60 (50,74)	74.5 (64.25,74.50)	0.062
Sex			0.215
Male	6	22	
Female	1	18	
Hepatic dysfunction	1 (14.3%)	12 (30%)	0.391
Rash	6 (85.7%)	29 (72.5%)	0.459
BCG scar reactivation	0	21 (52.5%)	0.012
Oropharyngeal changes	5 (71.4%)	32 (80%)	0.609
Conjunctival hyperemia	1 (14.3%)	29 (72.5%)	0.006
Peripheral limb alterations	2 (28.6%)	20 (50%)	0.423
Cervical lymphadenopathy	2 (28.6%)	23 (57.5%)	0.228
Perianal erythema/desquamation	0	5 (12.5%)	1.000
Cough	4 (57.1%)	37 (92.5%)	0.035
Nasal congestion and rhinorrhea	3 (42.9%)	19 (47.5%)	1.000
Vomiting	1 (14.3%)	6 (15%)	0.961
Diarrhea	4 (57.1%)	19 (47.5%)	0.701
Coronary artery lesion	6 (85.7%)	16 (40%)	0.040
Coronary artery aneurysm	5 (71.4%)	10 (25%)	0.026
cKD: iKD	1:6	23:17	0.048
Timing of initial IVIG administration (days)	4 (4, 5)	5.5 (4.25, 6.00)	0.155
Duration of fever (days)	7 (7, 12)	6 (5, 6)	0.006
Days of fever at diagnosis	4 (4, 5)	5 (4, 6)	0.153
White blood cell count (×10^9^/L)	12.43 ± 5.28	14.44 ± 4.76	0.316
Hemoglobin (g/L)	98 (80, 99)	97.5 (88, 105)	0.188
Mean platelet volume (fl)	9.24 ± 1.30	9.75 ± 1.18	0.237
Absolute neutrophil count (×10^9^/L)	7.31 (4.43, 9.91)	7.84 (5.51, 9.51)	0.654
Neutrophil percentage (%)	63.20 ± 13.99	53.91 ± 11.90	0.070
Platelet count (×10^9^/L)	511.57 ± 136.01	404.05 ± 166.14	0.113
NLR	2.59 (1.14, 4.41)	1.75 (1.12, 2.10)	0.054
PLR	203.88 (130.11, 244.08)	96.31 (58.99, 115.20)	0.002
SII (×10^9^)	1,658.99 (576.4, 2,818.45)	600.36 (408.55, 969.80)	0.027
Hematocrit (L/L)	0.283 ± 0.023	0.296 ± 0.030	0.287
C-reactive protein (mg/L)	85.94 ± 49.18	78.27 ± 49.42	0.570
Alanine aminotransferase (U/L)	17.6 (11.3, 22.1)	22.45 (17.65, 41.30)	0.071
Albumin (g/L)	38.99 ± 2.59	38.67 ± 2.77	0.781
Prealbumin (mg/L)	84.0 (65, 87)	79.5 (60.5, 90.0)	0.765
CAR	2.19 ± 1.24	2.05 ± 1.34	0.731
Cardiac troponin T (pg/ml)	22.01 (18.46, 44.66)	17.72 (11.68, 24.00)	0.078
Creatine kinase-MB (U/L)	3.50 (2.0, 4.20)	2.05 (1.40, 2.78)	0.094
LCR	310.26 (181.54, 1,401.57)	696.13 (408.17, 1,096.63)	0.142

IVIG, intravenous immunoglobulin; BCG, Bacille Calmette-Guérin; cKD, complete Kawasaki disease; iKD, incomplete Kawasaki disease; NLR, neutrophil-to-lymphocyte ratio; PLR, platelet-to-lymphocyte ratio; SII, systemic immune-inflammation index; LCR, lymphocyte count multiplied by 10,000 divided by CRP; CAR, C-reactive protein-to-albumin ratio.

The incidences of CAL, CAA, and iKD were significantly higher in the IVIG-resistant group than in the IVIG-responsive group (*P* < 0.05), whereas the incidences of BCG site reactivation, conjunctival injection, and cough were significantly lower in the IVIG-resistant group (*P* < 0.05). Laboratory data showed that PLR and SII were significantly higher in the IVIG-resistant group compared to the IVIG-responsive group (*P* < 0.05). The duration of fever was also significantly longer in the IVIG-resistant group (*P* < 0.05).

**Figure 1:**
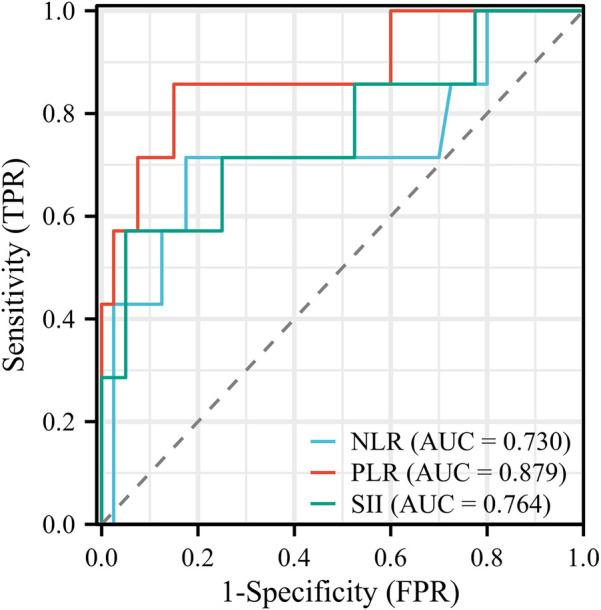
ROC curves for predicting IVIG resistance in infants with KD using NLR, PLR, and SIII. IVIG, intravenous immunoglobulin; KD, Kawasaki disease; NLR, neutrophil-to-lymphocyte ratio; PLR, platelet-to-lymphocyte ratio; SII, systemic immune-inflammation index.

Logistic regression analysis was performed on variables with significant differences and clinical predictive value. Elevated PLR was identified as an independent risk factor for IVIG resistance (OR: 1.029; 95% CI: 1.006–1.053; *P* = 0.013). According to the principle of maximizing the Youden index (Sensitivity + Specificity-1), the cut-off value for predicting IVIG resistance by the PLR is 124.05. Using a cut-off point of PLR ≥ 124.050, we could identify the IVIG resistant group with 85.71% sensitivity and 85.00% specificity. The ROC curve shows that the area under the curve (AUC) is 0.879, with a 95% CI of 0.712–1,000, see Figure 1 and Table 3 for details. Additionally, we constructed and compared ROC curves for the prediction of IVIG resistance in infants with KD using NLR, PLR, and SII, as detailed in Figure 1 and Table 3. We found that PLR had the largest area under the ROC curve for predicting IVIG resistance in infants with KD.

**Table 3:** ROC curves for predicting IVIG resistance in infants with KD using NLR, PLR, and SIII.

Characteristics	AUC	95% CI	Cut-off	Sensitivity	Specificity
NLR	0.730	0.472–0.989	2.305	71.43%	82.50%
PLR	0.879	0.712–1.000	124.050	85.71%	85.00%
SII	0.764	0.532–0.997	1,438.9	57.14%	95.00%

IVIG, intravenous immunoglobulin; KD, Kawasaki disease; NLR, neutrophil-to-lymphocyte ratio; PLR, platelet-to-lymphocyte ratio; SII, systemic immune-inflammation index.

### Prognosis and follow-up

All 47 patients were followed up for coronary artery involvement. A total of 22 patients developed CAL, with 19 cases occurring within the first 10 days and 3 cases developing after 10 days. Among patients who initially showed no CAL at diagnosis, 1 case developed CAL after 6 days of treatment and another after 5 days of treatment. Fifteen patients developed coronary artery aneurysms. Two patients were lost to follow-up, while the remaining 20 patients showed complete resolution of coronary lesions. Most patients achieved normalization of coronary arteries within 6 months after discharge, with the latest recovery observed at 16.2 months post-discharge.

## Discussion

This study systematically analyzed clinical features and IVIG resistance risk factors in KD infants younger than 3 months. Atypical clinical presentations were common, with a high iKD proportion, leading to diagnostic delays. CAL and IVIG resistance rates were significantly higher in iKD, emphasizing the greater cardiovascular risk in this age group. Elevated PLR were independent predictors of IVIG resistance, consistent with prior reports, underscoring the diagnostic and therapeutic challenges in very young KD patients.

KD is a systemic vasculitis predominantly affecting children, with its incidence showing notable regional and racial differences. The annual incidence in children under 5 years old in the United States is approximately 18–25 per 100,000, while in East Asian countries such as China, Japan, and South Korea, the incidence is 10–30 times higher (1). In China, the incidence ranges from 7.06 to 55.1 per 100,000 and continues to rise (2). The incidence of KD in infants younger than 3 months is significantly lower than in older children, and neonatal KD remains extremely rare. Large epidemiological studies from Korea and Spain have reported that infants younger than 3 months accounted for only 2.2% and 1.13% of KD cases, respectively (7, 8), consistent with the 1.91% observed in our study. A Korean study from Kyungpook National University Children's Hospital reported an incidence of approximately 4% (9). A Chinese study by Li et al. (24) found that infants under 3 months accounted for 3.48% (40/1,150) of KD cases. Despite its rarity, the risk of cardiovascular complications in this age group is markedly higher. The incidence of CAL in Chinese pediatric KD patients is as high as 15.9%, with CAA incidence at 1.8% (10, 11). Similar to previous studies, our results showed that atypical clinical manifestations, including shorter fever duration and less prominent mucocutaneous changes, frequently lead to delayed diagnosis and increase the risk of CAL and giant aneurysms.

The exact pathogenesis of KD remains unclear, though it is generally believed to be related to abnormal immune responses triggered by infection. Due to immature immune system development in infants younger than 3 months, their clinical manifestations are even more subtle. The AHA guidelines recommend that febrile infants who have fever lasting more than 7 days should be evaluated for KD even in the absence of classic features (1). In our study, iKD patients had lower rates of BCG site reactivation, conjunctival injection, oropharyngeal changes, extremity changes, and lymphadenopathy compared to cKD patients, further confirming the diagnostic difficulty in this age group. The steroid usage rate in the iKD group was higher than that in the cKD group, this is considered to be related to the possible occurrence of IVIG non-response due to delayed diagnosis in the iKD group. Special attention should be given to febrile infants with BCG site changes, which may serve as an important diagnostic clue (9). The proportion of iKD in our cohort reached 48.9%, much higher than the previously reported 19.4%–28.4% in China (12, 13), likely due to the younger age of our cohort. The incidence of CAL was 46.8% in our study, significantly higher than the 25% incidence reported in the 6th edition of the Japanese guidelines (14), but consistent with the 48.2% reported by Li Xueqin et al. for infants under 6 months (15). The incidence of CAL in iKD (65.2%) was significantly higher than in cKD (29.2%), consistent with previous studies suggesting a closer association between iKD and CAL development (15–17). This may be related to delayed diagnosis and missed optimal IVIG treatment windows in patients with atypical presentations, underscoring the importance of early IVIG administration in infants. Furthermore, some patients developed CAL within two weeks of onset even after IVIG initiation, highlighting the need for continuous coronary monitoring during the acute phase. In our cohort, the IVIG resistance rate was 14.9%, consistent with previous reports of 10%–20% (1). Among IVIG-resistant patients, 85.7% developed CAL, which was significantly higher than the 40% in responders, emphasizing IVIG responsiveness as a prognostic factor. Therefore, strengthening early identification and dynamic coronary monitoring in young infants is crucial to prevent disease progression.

In addition to atypical clinical manifestations, laboratory indicators in infants younger than 3 months also lack diagnostic specificity, increasing the difficulty of clinical recognition. In our study, only PLR and SII showed significant differences between IVIG responders and non-responders, while other commonly used inflammatory indicators were not predictive, suggesting the limited value of conventional laboratory parameters in this population, likely due to their immature immune response. Therefore, KD diagnosis in young infants still requires comprehensive clinical assessment and dynamic coronary evaluation. Some studies have suggested seasonal variation in KD incidence, with peaks in spring and summer in China (16, 25). A 15-year Japanese study found that over 60% of KD cases in infants younger than 4 months occurred in summer and autumn (18). In our study, 61.7% of KD cases occurred in spring and autumn, suggesting that seasonal distribution may be affected by geography, climate, and circulating pathogens. Larger multicenter studies are needed to clarify these epidemiological trends and mechanisms.

The early use of steroids in KD remains controversial, as they are typically reserved for cases where IVIG treatment is ineffective. At our hospital, steroids are primarily used for IVIG-resistant cases or patients with more severe symptoms. This approach aligns with current guidelines and recommendations (19), which emphasize that steroid use should be based on individual circumstances, rather than being a routine treatment, especially in younger infants. Similarly, dual antiplatelet therapy is not routinely administered to all KD patients. According to current guidelines and clinical practice (1), it is generally reserved for IVIG-resistant cases, patients with severe coronary artery involvement, or those with recurrent episodes where standard treatments are ineffective. These patients often require more potent antiplatelet effects due to poor responses to conventional treatments. Before initiating dual antiplatelet therapy, specialists must carefully evaluate its potential benefits and risks, ensuring it is used with caution.

Our logistic regression analysis identified PLR ≥124.05 as an independent risk factor for IVIG resistance. This is partly consistent with Kanai et al.'s findings on PLR (20), but differs from Chen et al. (21) and Wu et al. (22) who emphasized the predictive value of neutrophil-to-lymphocyte ratio (NLR). Such differences may be attributed to younger patient age (<3 months), limited sample size, and regional variations. Although Yi et al. (23) first proposed that SII ≥2,209.66 × 10⁹ could predict IVIG resistance, we did not observe predictive value for SII in our cohort. Possible explanations include immature immune responses (6), insignificant ANC and PLT differences between responder groups, and small sample size. Further large-scale studies are warranted to verify the predictive value of SII in this population.

This study offers valuable insights into the clinical characteristics and risk factors for IVIG resistance in infants under 3 months of age with Kawasaki disease (KD). However, several limitations should be noted. Firstly, infants who received IVIG prior to admission were excluded to minimize confounding factors and ensure a more homogeneous study population. While this decision strengthens the internal validity, it may reduce the representativeness of the sample and limit the generalizability of the findings. Secondly, the retrospective, single-center design with a limited sample size introduces potential selection bias and further restricts the generalizability. Although PLR was identified as a potential predictor, we did not compare its performance with existing scoring systems or establish a localized risk model, which limits its clinical applicability. Additionally, the small sample size of 47 infants, including only 7 IVIG-resistant cases, reduces statistical power and increases the risk of overfitting in the multivariate regression model. The wide confidence intervals observed suggest instability in the model's estimates. While cross-validation might address this, the small sample size complicates its implementation. Therefore, larger cohort studies are necessary to confirm these findings and strengthen the conclusions. Future prospective, multicenter studies incorporating larger sample sizes and immunologic biomarkers (such as cytokines and molecular markers) are essential for developing early risk prediction models. Such models could ultimately improve the precision and timeliness of diagnosis and management for this vulnerable population.

## Data Availability

The original contributions presented in the study are included in the article/Supplementary Material, further inquiries can be directed to the corresponding author.

## References

[1] Jone P Tremoulet A Choueiter N Dominguez SR Harahsheh AS Mitani Y Update on diagnosis and management of Kawasaki disease: a scientific statement from the American Heart Association. Circulation. (2024) 150:e481–500. 10.1161/CIR.0000000000001295 39534969

[2] Elakabawi K Lin J Jiao F Guo N Yuan Z. Kawasaki disease: global burden and genetic background. Cardiol Res. (2020) 11:9–14. 10.14740/cr993 32095191 PMC7011927

[3] Faim D Henriques C Brett A Francisco A Rodrigues F Pires A. Kawasaki disease: predictors of resistance to intravenous immunoglobulin and cardiac complications. Arq Bras Cardiol. (2021) 116:485–91. 10.36660/abc.20190758 33470332 PMC8159558

[4] Fukazawa R Kobayashi J Ayusawa M Hamada H Miura M Mitani Y JCS/JSCS 2020 guideline on diagnosis and management of cardiovascular sequelae in Kawasaki disease. Circ J. (2020) 84:1348–407. 10.1253/circj.CJ-19-1094 32641591

[5] Son MBF Gauvreau K Newburger JW. Failure of risk prediction modeling for IVIG resistance in Kawasaki disease. Pediatrics. (2023) 151(5):e2022060423. 10.1542/peds.2022-060423 37092273

[6] Kim DS. Kawasaki disease. Yonsei Med J. (2006) 47:759–72. 10.3349/ymj.2006.47.6.759 17191303 PMC2687814

[7] Lee EJ Park YW Hong YM Lee JS Han JW. Epidemiology of Kawasaki disease in infants 3 months of age and younger. Korean J Pediatr. (2012) 55:202–5. 10.3345/kjp.2012.55.6.202 22745644 PMC3382700

[8] Grasa CD Fernandez-Cooke E Sanchez-Manubens J Anton J Crespo D Garcia M Kawasaki disease in infants 3 months of age and younger: a multicentre Spanish study. Ann Rheum Dis. (2019) 78:289–90. 10.1136/annrheumdis-2018-213891 30282667

[9] Roh DE Kwon JE Kim YH. Bacille calmette-guerin site reactivation of Kawasaki disease in infants under 3 months of age: relation with diagnosis and prognosis. Children (Basel). (2022) 9(6):857. 10.3390/children9060857 35740793 PMC9222052

[10] Ramos M Seguro Paula F Carvalho A Pinheiro M Ramos A. Kawasaki disease: a rare case of a non-pediatric patient. Cureus. (2024) 16:e74824. 10.7759/cureus.74824 39737277 PMC11684353

[11] Mu Z Jiao F Xie K. Interpretation of “diagnosis and management guidelines for cardiovascular sequelae in Kawasaki disease (JCS/JSCS 2020)”. Chin J Contemp Pediatr. (2021) 23:213–20. 10.7499/j.issn.1008-8830.2010134 PMC7969191 33691912

[12] Xu M Zou Y Liang Y Cai J Liu R. Clinical significance of peripheral blood NLR, PLR and SII in infantile Kawasaki disease. Chin J Woman Child Health Res. (2025) 36:78–84.

[13] Jiao F Pan Y Du Z Deng F Yang X Wang H Guideline for the diagnosis and treatment of incomplete Kawasaki disease in children in China. BMC Pediatr. (2024) 24:477. 10.1186/s12887-024-04961-2 39060924 PMC11282762

[14] Kobayashi T Ayusawa M Suzuki H Abe J Ito S Kato T Revision of diagnostic guidelines for Kawasaki disease (6th revised edition). Pediatr Int. (2020) 62:1135–38. 10.1111/ped.14326 33001522

[15] Li X Wang J Wang X Zhao A Gu H Zhang H Characteristics and risk factors of coronary artery lesions in infants under 6 months with Kawasaki disease. Lab Med Clin. (2022) 19:2177–80.

[16] Shi L Li J Qie D Hua X Pan J Shi X Clinical manifestations of Kawasaki disease in different age groups: retrospective data from southwest China. Clin Rheumatol. (2020) 39:3027–32. 10.1007/s10067-020-05069-5 32367406

[17] An HS Kim GB Song MK Lee SY Kwon HW Lee JW The occurrence of coronary artery lesions in Kawasaki disease based on C-reactive protein levels: a retrospective cohort study. Pediatr Rheumatol Online J. (2021) 19:78. 10.1186/s12969-021-00566-6 34078404 PMC8173749

[18] Kitano N Suzuki H Takeuchi T. Patient age and the seasonal pattern of onset of Kawasaki’s disease. New Engl J Med. (2018) 378:2048–9. 10.1056/NEJMc1804312 29791820

[19] McCrindle BW Rowley AH Newburger JW Burns JC Bolger AF Gewitz M Diagnosis, treatment, and long-term management of Kawasaki disease: a scientific statement for health professionals from the American Heart Association. Circulation. (2019) 140:e181–84. 10.1161/CIR.0000000000000703 28356445

[20] Kanai T Takeshita S Kawamura Y Kinoshita K Nakatani K Iwashima S The combination of the neutrophil-to-lymphocyte and platelet-to-lymphocyte ratios as a novel predictor of intravenous immunoglobulin resistance in patients with Kawasaki disease: a multicenter study. Heart Vessels. (2020) 35:1463–72. 10.1007/s00380-020-01622-z 32449049

[21] Chen Y Hua Y Zhang C Chen S Zhang Q Liao Y Neutrophil-to-Lymphocyte ratio predicts intravenous immunoglobulin-resistance in infants under 12-months old with Kawasaki disease. Front Pediatr. (2019) 7:81. 10.3389/fped.2019.00081 30941338 PMC6433842

[22] Wu S Long Y Chen S Huang Y Liao Y Sun Y A new scoring system for prediction of intravenous immunoglobulin resistance of Kawasaki disease in infants under 1-year old. Front Pediatr. (2019) 7:514. 10.3389/fped.2019.00514 31921727 PMC6917618

[23] Yi C Zhou Y Guo J Chen J She X. Novel predictors of intravenous immunoglobulin resistance in patients with Kawasaki disease: a retrospective study. Front Immunol 2024;15:1399150. 39040113 10.3389/fimmu.2024.1399150 PMC11260624

[24] Li W Zhang L Huang P Zhang Z. Clinical features and mid-term follow-up in infants younger than 3 months with Kawasaki disease in a Chinese population. J Paediatr Child H. (2019) 55:523–7. 10.1111/jpc.14233 30246351

[25] Liu HH Qiu Z Fan GZ Jiang Q Li RX Chen WX Assessment of coronary artery abnormalities and variability of Z-score calculation in the acute episode of Kawasaki disease-A retrospective study from China. Eur J Clin Invest. (2021) 51:e13409. 10.1111/eci.13409 32916764

